# Studying medical communication with video vignettes: a randomized study on how variations in video-vignette introduction format and camera focus influence analogue patients’ engagement

**DOI:** 10.1186/s12874-018-0472-3

**Published:** 2018-01-19

**Authors:** Leonie N. C. Visser, Nadine Bol, Marij A. Hillen, Mathilde G. E. Verdam, Hanneke C. J. M. de Haes, Julia C. M. van Weert, Ellen M. A. Smets

**Affiliations:** 1 Department of Medical Psychology, Academic Medical Center, University of Amsterdam, P.O. Box 22660, 1100 DD Amsterdam, the Netherlands; 2 Amsterdam School of Communication Research/ASCoR, University of Amsterdam, Amsterdam, The Netherlands; 3 Research Institute of Child Development and Education, University of Amsterdam, Amsterdam, The Netherlands; 4 Department of Methodology and Statistics, Institute of Psychology, Leiden University, Leiden, The Netherlands

**Keywords:** Patient-provider communication, Video-vignettes design, Analogue patients, Camera viewpoint, Introduction, Engagement, Psychophysiology

## Abstract

**Background:**

Video vignettes are used to test the effects of physicians’ communication on patient outcomes. Methodological choices in video-vignette development may have far-stretching consequences for participants’ engagement with the video, and thus the ecological validity of this design. To supplement the scant evidence in this field, this study tested how variations in video-vignette introduction format and camera focus influence participants’ engagement with a video vignette showing a bad news consultation.

**Methods:**

Introduction format (A = audiovisual vs. B = written) and camera focus (1 = the physician only, 2 = the physician and the patient at neutral moments alternately, 3 = the physician and the patient at emotional moments alternately) were varied in a randomized 2 × 3 between-subjects design**.** One hundred eighty-one students were randomly assigned to watch one of the six resulting video-vignette conditions as so-called analogue patients, i.e., they were instructed to imagine themselves being in the video patient’s situation. Four dimensions of self-reported engagement were assessed retrospectively. Emotional engagement was additionally measured by recording participants’ electrodermal and cardiovascular activity continuously while watching. Analyses of variance were used to test the effects of introduction format, camera focus and their interaction.

**Results:**

The audiovisual introduction induced a stronger blood pressure response during watching the introduction (*p* = 0.048, ${\eta}_{partial}^2$= 0.05) and the consultation part of the vignette (*p* = 0.051, ${\eta}_{partial}^2$= 0.05), when compared to the written introduction. With respect to camera focus, results revealed that the variant focusing on the patient at emotional moments evoked a higher level of electrodermal activity (*p* = 0.003, ${\eta}_{partial}^2$= 0.06), when compared to the other two variants. Furthermore, an interaction effect was shown on self-reported *emotional* engagement (*p* = 0.045, ${\eta}_{partial}^2$= 0.04): the physician-only variant resulted in lower emotional engagement if the vignette was preceded by the audiovisual introduction. No effects were shown on the other dimensions of self-reported engagement.

**Conclusions:**

Our findings imply that using an audiovisual introduction combined with alternating camera focus depicting patient’s emotions results in the highest levels of *emotional* engagement in analogue patients. This evidence can inform methodological decisions during the development of video vignettes, and thereby enhance the ecological validity of future video-vignettes studies.

## Background

High-quality communication is an essential requirement for physicians when conducting medical consultations. It enables physicians, for example, to adequately provide information to, and gather information from, patients [1]. The growing interest in research investigating how to optimize medical communication is therefore not surprising. However, solid evidence on how physicians’ communication impacts patient outcomes, such as satisfaction or information recall, is still scarce. This may be because causal relationships between specific aspects of communication and outcomes are difficult to prove in actual medical practice, as these relations are influenced by many (confounding) variables. To overcome this barrier and provide solid evidence, an experimental video-vignettes design can be used.

A video-vignettes design requires the development of multiple (brief) videos of a scripted medical consultation [2]. The key to this design is standardization: the communication, medical content, and appearance of the environment and the characters are kept exactly identical across videos except for the communication elements of interest. By varying, i.e. manipulating, specific verbal or non-verbal communication elements across conditions while keeping the remainder constant, we can study the effectiveness of specific elements in physicians’ communication. The video vignettes are viewed by study participants, who can be either disease-naive (‘healthy’) individuals or patients with a medical history regarding the disease in the video vignette. These so-called analogue patients are instructed to imagine themselves being in the shoes of the patient in the video. Consequently, (analogue) patient outcomes can be easily assessed without needing to manipulate or intrude on actual medical consultations.

Previous research has indicated that analogue patients can be validly used as proxies to actual patients to evaluate physicians’ communication behavior [3, 4]. However, a prerequisite to the ecological validity of the video-vignettes design, i.e., the extent to which the act of watching a video vignette as an analogue patient resembles a patient’s experience in actual medical practice, is that analogue patients sufficiently engage in the video. Video engagement is a multidimensional construct which can be defined as the degree to which analogue patients view the video attentively, submerse in the video vignette’s story, identify with the video patient, and experience empathy and emotions [5]. The level of analogue patients’ engagement is likely to depend on how the videos are developed. Many methodological variations are possible, yet little empirical data is available on the potential consequences of such variations on (analogue) patient outcomes [2, 6].

Two of the most salient methodological issues with regard to the video-vignettes design are how the video vignettes are introduced and which focus is used to show the medical consultation. Because these two issues may crucially influence analogue patients’ engagement when viewing video vignettes, they are the focus of the current study. An introduction to the video vignette can be added to familiarize analogue patients to the setting and the characters, inform analogue patients about the video patient’s situation, instruct, and/or engage analogue patients. Various introduction formats may be used. For example, the researcher could show a written introduction on paper or a (computer) screen or provide an audiovisual introduction, i.e., showing video images with a neutral voice-over or a videotaped scene in which the patient introduces his−/herself [2]. Likewise, several variations are possible when considering camera focus. Since the physician’s communication is under investigation in most video-vignette studies [2, 6], it is essential for analogue patients to be able to observe the communication behavior of the physician depicted in the video vignette. The focus on the physician in the video vignette is thus a logical consequence, which can be effectuated by using camera shot types facing the physician, such as (medium) close-ups of the physician, or over-the-shoulder-of-the-patient shots. Alternatively, to enable analogue patients to better observe the (natural course of the) interaction between the physician and the patient, one could decide to incorporate camera shots depicting the patient’s communication behavior (as well) [2]. Broadly considered, two choices are therefore available with regard to camera focus, i.e., to focus on the physician only, or to alternate camera focus between the physician and the patient. Furthermore, in case of the latter, the amount of time and the specific moments that the patient is shown can be varied and might influence analogue patients’ engagement with the video vignette.

Although no evidence exists regarding the effects of introduction format or camera focus on video-vignette engagement, some hypotheses can be put forward based on literature from other fields. With regard to introduction format, engagement might be highest when stimulating multiple sensory modalities [7]. An audiovisual introduction is therefore expected to have the strongest engagement-inducing effect when compared to an introduction provided only visually, such as a written introduction. With regard to camera focus, alternating between the physician and the patient might increase attentional engagement as a mere result of analogue patients’ responding to novelty or change, i.e., the so-called orienting response [8]. Furthermore, research suggests that observing another person’s emotional expressions stimulates feelings of emotional empathy through mirror neuron activity [9], and it is known that emotional faces attract more attention than neutral faces [10]. This indicates that focusing on the video patient specifically when expressing emotions can enhance emotional and attentional engagement even more.

The aim of this study was to systematically investigate the effects of introduction format and camera focus on analogue patients’ video engagement. Firstly, analogue patients’ engagement was expected to be the strongest when watching a video vignette with an audiovisual introduction, when compared to a written introduction. Secondly, alternating the camera focus between the physician and the video patient was expected to cause a higher level of engagement, as compared to a physician-only variant. More specifically, alternating the camera focus depicting the video patient’s emotional expressions, was expected to cause higher levels of engagement than an alternating variant that focusses on the patient at relatively neutral moments, and a physician-only variant. Thirdly, combining an audiovisual introduction and alternating camera focus depicting the patient’s emotional expressions, was expected to induce the highest levels of engagement.

## Methods

### Design and ethics statement

**Fig. 1:**
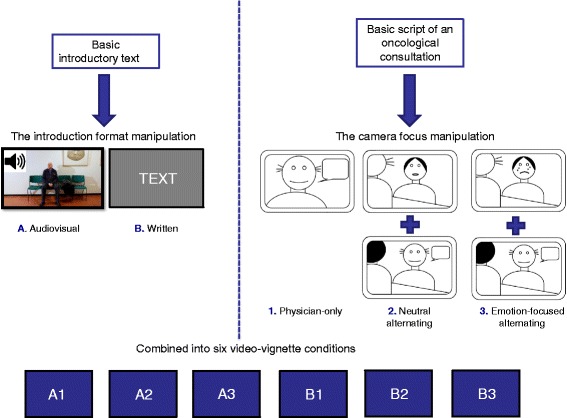
Visual illustration of the development of the six video-vignette conditions

This experimental video-vignettes study used a videotaped scripted medical consultation in which two methodological elements (introduction format and camera focus) were varied in a randomized 2 by 3 between-subjects design, resulting in six video-vignette conditions (see Fig. 1). Video engagement was operationalized in two ways. First, four dimensions of engagement were measured by means of self-report. Because of its retrospective nature, relying on self-report only might be prone to bias. Therefore, multiple so-called psychophysiological measures were additionally used to make direct inferences about analogue patients’ *emotional* engagement during watching the introduction and the medical consultation part of the video vignette [11, 12]. The standardized video vignettes depicted an oncological bad news consultation to enable a strong emotional response. The study protocol was approved by the Ethics Committee of the department of Communication Science at the University of Amsterdam (2014-CW-30). Written informed consent was obtained from all participants.

### Sample

Cancer-naive individuals aged between 18 and 40 were recruited as analogue patients through a Psychology and a Communication Science research eduation program of the University of Amsterdam. Participants were eligible if they: 1) were not using medication related to cardiovascular disease or hypertension (to prevent interference with psychophysiological measures of engagement); 2) were literate in Dutch; 3) had no prior experience with oncological consultations as a patient or a patient’s relative.

### Video vignettes

The scripted video vignettes were previously developed as part of a research line addressing understanding and improving physician-patient information transfer within the field of oncology. The video vignettes showed a bad news consultation involving an oncological surgeon (referred to as ‘the physician’ in this manuscript) and a patient discussing the patient’s cancer diagnosis, prognosis, treatment options and side-effects. Two identical versions were created for each video-vignette condition, one with a male and one with a female actor in the role of the video patient. By matching analogue patients’ gender to that of the video patient, identification was optimized. A detailed description of the developmental process of the video vignettes used in the current study can be found in a previous publication (see [12]).

#### The introduction manipulation: format A & B

An introduction preceded the video vignette to present the video patient and his/her medical situation. Two formats were developed (see Fig. 1): *A) an audiovisual introduction*, depicting a video fragment of the patient in the waiting area of a hospital while a neutral voice-over reads the introductory text out loud; *B) a written introduction*, in which the same introductory text appears on the screen in five successive parts (each showing approximately 9 s) in white letters on a grey background. The duration of both introduction formats was 49 s.

#### The camera focus manipulation: variant 1 to 3

The medical consultation depicted in the video vignette was videotaped using multiple cameras. From these recordings, three camera focus variants were developed during editing (see Fig. 1): *1) physician-only*: focusing on the physician during the entire medical consultation; *2) neutral alternating:* alternating camera focus between the physician and the patient, focusing on the patient at relatively neutral moments in the consultation; *3) emotion-focused alternating*: alternating camera focus between the physician and the patient, focusing on the patient at emotional moments in the consultation. For variant 1 only (medium) close-up shots of the physician were used. Variant 2 and 3 were created by combining multiple camera shot types, i.e., by means of (medium) close-up and over-the-shoulder shots, facing the physician or the patient. The video-vignette variants varied slightly in length, between 368 and 386 s, because of small editing differences across variants, and small differences in speaking rate between the male and female video-patient versions.

### Experimental procedures

A detailed description of experimental procedures and measures was previously published as part of a separate study on the relationship between (analogue) patients’ emotional stress response and memory of medical information [13]. Here, we provide a summary of information relevant for the current study.

Upon arrival in a standardized laboratory room, analogue patients completed a digital questionnaire (T0) reporting their age, gender, weight and height (the latter two to calculate their Body Mass Index (BMI), which is known to correlate with physiological activity [12]). They were then attached to the psychophysiological equipment. Analogue patients first watched a fragment of a calm nature documentary on a computer screen to get acclimatized and determine psychophysiological baseline values (eight and three minutes respectively). Next, they were randomly assigned to one of the six video-vignette conditions (see Fig. 1). Psychophysiological activity was recorded continuously while analogue patients watched both the introduction and the consultation part of the video vignette. Immediately after watching, they completed a second digital questionnaire (T1) to assess their engagement with the video and their perception of video realism. Psychophysiological equipment was then removed and analogue patients were awarded one research program credit or ten euros for their participation.

### Measures

#### Engagement

Self-reported engagement with the video vignette was assessed using the 15-item Video Engagement Scale (VES) [5]. This questionnaire was specifically developed and validated to retrospectively assess analogue patients’ video engagement. Four dimensions of engagement were assessed: (1) Emotions and Empathy, emotional reactions evoked by the video and empathy with the video patient (EMP/EMO; Cronbach’s alpha = 0.91); (2) Identity, adopting the video patient’s identity (IDE; Cronbach’s alpha = 0.92); (3) Attention, attentional focus on the video (ATT; Cronbach’s alpha = 0.66), and (4) Going into a Narrative World, the sensation of being immersed in the story and world shown in the video vignette (GNW; Cronbach’s alpha = 0.88). All items were answered on a 7-point Likert scale (1 = ‘completely disagree’ to 7 = ‘completely agree’). Mean scores on the individual dimensions were used in analyses.

Since physiological activity varies as a function of psychological change, psychophysiological measurement can be used to make inferences about psychological processes, in particular analogue patients’ emotional arousal as evoked by watching the video vignette [11]. Multiple psychophysiological measures were assessed in this study to increase sensitivity to detect effects, as it is argued that the emotional response has specific patterns of peripheral physiological activation in response to different emotions [14]. Electrodermal activity (EDA) refers to changes in the electrical properties of the human skin in response to sweat secretion. EDA includes a slowly varying tonic component (skin conductance level: SCL) and a fast-changing phasic component (skin conductance responses: SCRs). EDA was recorded continuously during the baseline period, the introduction, and the consultation part of the video vignette. The wireless BioNomadix EDA module (Biopac) was used with two disposable electrodes attached to the index and middle finger of the right hand. A low pass filter fixed at 1 Hz was used to eliminate any high frequency noise from the signal. The mean level of EDA in micro Siemens (μS), from now on referred to as SCL [15], was calculated using AcqKnowledge software. SCRs were also identified (using a threshold of 0.05 μS) and the number of SCRs per minute was calculated [15]. Cardiovascular activity was additionally assessed using three parameters, i.e., systolic blood pressure (SBP, in mm/Hg), diastolic blood pressure (DBP, in mm/Hg), and heart rate (HR, in beats per minute (BPM)). A Finometer Pro (Finapres Medical Systems) was connected to the Biopac system to continuously record cardiovascular activity while watching the video. The Finometer Pro cuff was connected to the ring finger of analogue patients’ left hand. The values of SBP and DBP were generated per heartbeat and filtered with a low pass filter fixed at 35 Hz. HR was calculated from the blood pressure signal. Mean activity levels were calculated using AcqKnowledge.

#### Perceived realism

Perceived realism of the video vignette, i.e., the extent to which the video-vignettes’ characters and situations are judged to be similar to real life, was assessed for exploratory purposes. This construct is thought to be related to engagement [16, 17] and might impact the ecological validity of the video-vignette design as well. Perceived realism was assessed by asking how realistic and likely to happen in real life the analogue patients found 1) the events in the video vignette (3 items; Cronbach’s alpha = 0.84); 2) the behavior and appearance of the patient in the video (2 items; Cronbach’s alpha = 0.79); and 3) the physician’s behavior and appearance (2 items; Cronbach’s alpha = 0.78). These items were previously developed for video-vignette research by our group (see for example [12]). All items were answered on a 7-point Likert scale (1 = ‘completely disagree’ to 7 = ‘completely agree’). Mean scores were calculated for these three scales.

### Data processing and statistical analyses

All analyses were performed using IBM SPSS Statistics 23. The 0.05 probability level was used as a criterion of statistical significance. Partial eta-squared (${\eta}_{partial}^2$*)* is reported as a measure of effect size. It describes the proportion of variance in the dependent variable attributable to a particular independent variable (and not explained by other independent variables) [18]. Suggested norms for partial eta-squared are: small = 0.01; medium = 0.06; large = 0.14 [19]. Self-reported data from one analogue patient were removed because these were judged to be unreliable based on the researcher’s log comments. Psychophysiological data of four analogue patients were not recorded due to equipment failure. Electrodermal data from 50 (28%), blood pressure data from 20 (11%), and heart rate data from 16 analogue patients (9%) were judged to be unreliable due to several severe signal disruptions (lasting for at least 5 s) and/or observed repeated movement of analogue patients. These data were therefore excluded from analyses. Normality of data distributions was evaluated using statistics (skewness/kurtosis) in conjunction with visual inspection. Log_10_ or square root transformations were applied on psychophysiological data. No outliers were removed.

In previous research, associations have been shown between analogue patients’ gender, age, BMI and outcomes [5, 20, 21]. Therefore, the Chi-square test statistic and ANOVAs were used to test for differences across conditions. Furthermore, MANOVAs and correlation analyses were used to check for associations between characteristics and outcome measures, as better estimations of the influence of introduction format and camera focus on outcomes might be obtained by adjusting for these characteristics (i.e., only if associations were found). Two 2 by 3 MANOVAs were used to test the influence of introduction format and camera focus on self-reported engagement and perceived realism. Repeated measures MANOVAs were used separately for cardiovascular and electrodermal psychophysiological parameters, to: 1) test the influence of introduction format on the change in analogue patients’ physiological activity from baseline to the video-vignette introduction, and 2) test the (combined) effects of introduction format and camera focus on the change in physiological activity from baseline to the video-vignette consultation. Sensitivity of the multivariate analyses, i.e., the MANOVAs, considering the resulting sample sizes (ranging from *N* = 127 to *N* = 180) was calculated using G-power 3.1.9.2, demonstrating 80% power to detect medium effects (ranging from partial eta-squared 0.04 to 0.07). The multivariate analyses were followed up by univariate analyses of variance to be able to examine the effects on the separate outcome measures. With regard to the psychophysiological parameters, follow up univariate results were only reported if significant effects were shown in multivariate analyses, to prevent type I error resulting from multiple testing.

## Results

### Analogue patients’ characteristics

Analogue patients (*N* = 181) were on average 23 years old (SD = 4.0; range 18–40), 70% were female (*N* = 126), and mean BMI was 22 (SD = 2.5). Randomization was successful as no differences between the six video-vignette conditions were found with regard to analogue patients’ gender (*p* = 0.541), age (*p* = 0.084), or BMI (*p* = 0.515). The following associations were shown between analogue patients’ characteristics and outcomes: 1) age was positively correlated with self-reported attentional engagement (ATT-dimension) during watching the vignette (*r* = 0.16; *p* = 0.036), and with blood pressure (systolic and diastolic) during all time periods (*r*-values ≥0.17; *p*-values ≤0.036); 2) men showed higher levels of diastolic blood pressure during all time periods (*p*-values ≤0.017); 3) BMI was positively correlated with skin conductance responses (SCRs) during the consultation part of the vignette (*r* = 0.18; *p* = 0.040). Based on these results, subsequent analyses were adjusted for age and gender when relevant. Electrodermal activity analyses were not adjusted for BMI, as BMI was only associated with SCRs during the consultation.

### The influence of introduction format and camera focus on self-reported engagement

**Fig. 2:**
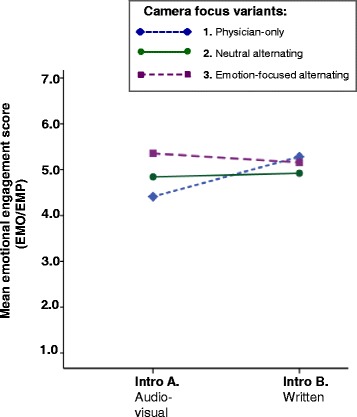
Analogue patients’ self-reported emotional engagement with the video vignette: an interaction effect of introduction format and camera focus. *Notes.* For analogue patients who watched the physician-only variant (variant 1), an effect of introduction format was found: the audiovisual introduction (format A) resulted in lower emotional engagement than the written introduction (format B; *p* = 0.004)

Table 1 shows mean scores and standard deviations of self-reported engagement stratified by introduction format and camera focus. Contrary to our expectations, no multivariate main effects were found of introduction format (Wilks’ Lambda = 0.98, *F*(4, 170) = 1.07, *p* = 0.375, ${\eta}_{partial}^2$= 0.02) or camera focus (Wilks’ Lambda = 0.96, *F*(8, 340) = 0.80, *p* = 0.605, ${\eta}_{partial}^2$= 0.02) on self-reported engagement with the video vignette. No multivariate interaction effect was found either (Wilks’ Lambda = 0.93, *F*(8, 340) = 1.56, *p* = 0.137, ${\eta}_{partial}^2$= 0.04). Furthermore, follow up univariate tests on the separate self-reported engagement dimensions only revealed a small to medium interaction effect on analogue patients’ score on the EMO/EMP dimension, i.e., analogue patients’ emotions in response to the vignette and empathy with the video patient (*p* = 0.045, ${\eta}_{partial}^2$= 0.04). For analogue patients who watched the physician-only variant (variant 1), an effect of introduction format was found, as shown in Fig. 2; the audiovisual introduction (format A) resulted in lower emotional engagement than the written introduction (format B; *p* = 0.004).

**Table 1:** Analogue patients’ self-reported engagement: means and standard deviations stratified by introduction format (A or B) and camera focus (1, 2 or 3)

Introduction format	Camera focus		ATT	GNW	IDE	EMO/EMP
		N	M (SD)	M (SD)	M (SD)	M (SD)
A. Audiovisual	1. Physician-only	30	5.41 (0.96)	4.25 (1.18)	3.53 (1.27)	**4.41** (1.10)
	2. Neutral alternating	31	5.17 (1.12)	4.36 (1.40)	3.54 (1.57)	4.84 (1.26)
	3. Emotion-focused alternating	31	5.26 (1.12)	4.55 (1.47)	3.91 (1.59)	5.35 (1.03)
	Total	92	5.28 (1.06)	4.39 (1.35)	3.66 (1.48)	4.88 (1.19)
B. Written	1. Physician-only	29	5.22 (1.10)	4.52 (1.22)	3.84 (1.38)	**5.28** (1.12)
	2. Neutral alternating	30	5.38 (0.96)	4.31 (1.26)	3.63 (1.57)	4.92 (1.28)
	3. Emotion-focused alternating	29	5.46 (4.34)	4.34 (1.07)	3.67 (1.29)	5.16 (1.16)
	Total	88	5.35 (1.01)	4.39 (1.18)	3.71 (1.41)	5.12 (1.19)
Total	1. Physician-only	59	5.32 (1.03)	4.38 (1.20)	3.68 (1.32)	4.84 (1.19)
	2. Neutral alternating	61	5.27 (1.04)	4.34 (1.32)	3.58 (1.56)	4.88 (1.26)
	3. Emotion-focused alternating	60	5.36 (1.05)	4.45 (1.28)	3.79 (1.45)	5.26 (1.09)
	Total	180	5.31 (1.03)	4.39 (1.26)	3.69 (1.44)	4.99 (1.19)

*Notes*. Possible range in values is 1–7; higher values indicate more engagement. Dimensions of engagement: ATT = attention; GNW = going into the narrative world; IDE = identity; EMO/EMP; emotions and empathy. The values in **bold** indicate an interaction effect on self-reported emotional engagement (EMO/EMP), as displayed in Fig. 2

### The influence of introduction format on the psychophysiological response during the video-vignette introduction

Means and standard deviations of analogue patients’ psychophysiological activity, including the observed response during the video-vignette introduction compared to baseline activity, are shown in Table 2 for the total sample of analogue patients.

**Table 2:** Analogue patients’ psychophysiological activity: total sample means, standard deviations and mean observed responses compared to baseline values

			Baseline		Introduction			Consultation		
	N	Range	M	SD	M	SD	M ∆	M	SD	M ∆
SBP (mm/Hg)	157	85.7–172.7	118.9	15.9	125.9	17.8	7.0	122.1	16.8	3.2
DBP (mm/Hg)	157	47.5–123.6	69.7	11.3	72.9	12.3	3.1	71.4	11.7	1.6
HR (BPM)	161	45.4–109.8	71.0	9.6	72.6	10.2	1.6	72.1	9.9	1.1
SCL (μS)	127	0.6–22.6	3.9	3.0	5.4	3.9	1.4	4.9	3.4	0.9
SCRs (spikes/min)	127	0–11	1.3	1.7	2.9	2.9	1.6	2.1	2.1	0.8

*Notes.* Original data are displayed in this table: psychophysiological activity during baseline, the introduction part of the vignette, and the consultation part of the vignette. Data are not stratified by introduction format and camera focus, i.e., this table displays the increase in psychological activity as elicited by watching the vignette for the total analogue patient sample. M ∆ = the mean observed response compared to baseline values; SBP = systolic blood pressure; DBP = diastolic blood pressure; HR = heart rate; SCL = skin conductance level; SCRs = skin conductance responses. Logarithms (square roots in case of SCRs) of the original values were used in analyses. Significant effects of introduction format and camera focus on the increase in psychophysiological activity from baseline to the vignette are displayed in Fig. 3a, b and 4

**Fig. 3:**
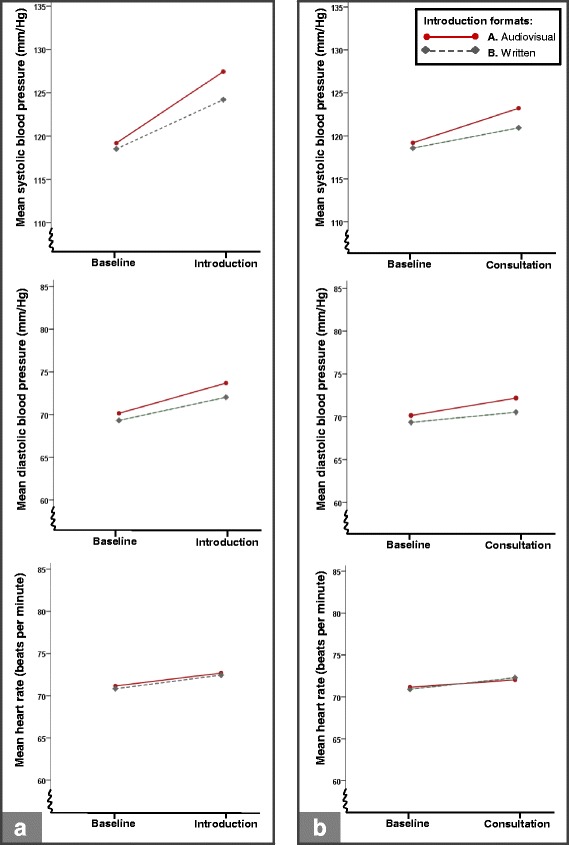
Impact of introduction format on the increase in cardiovascular activity from baseline to the video-vignette introduction (**a**) and consultation (**b**). *Notes.* This figure shows, from top to bottom, analogue patients’ mean systolic blood pressure, diastolic blood pressure and heart rate. Figure 3a, at the left, shows their cardiovascular activity at baseline and during the video-vignette *introduction*, stratified by introduction formats. Significant effect were shown on systolic blood pressure (*p* = 0.008) and diastolic blood pressure (*p* = 0.042), but not on heart rate (*p* = 0.896). Figure 3b, at the right, shows analogue patients’ mean cardiovascular activity at baseline and during the video-vignette *consultation*, stratified by introduction formats. Significant effect were shown on systolic blood pressure (*p* = 0.015) and diastolic blood pressure (*p* = 0.015), but not on heart rate (*p* = 0.314)

With regard to the *cardiovascular* parameters, a medium multivariate effect of introduction format was present; watching the audiovisual introduction (format A) evoked a stronger increase in cardiovascular activity in analogue patients (Wilks’ Lambda = 0.95, *F*(3, 150) = 2.70, *p* = 0.048, ${\eta}_{partial}^2$= 0.05) than watching the written introduction (format B). Follow up univariate results, illustrated in Fig. 3a, showed that analogue patients who watched the audiovisual introduction showed a larger blood pressure response, both systolic blood pressure (*p* = 0.008, ${\eta}_{partial}^2$= 0.05) and diastolic blood pressure (*p* = 0.042, ${\eta}_{partial}^2$= 0.03) than analogue patients who watched the written introduction. No effect on heart rate was shown (*p* = 0.896, ${\eta}_{partial}^2$< 0.01). With regard to the *electrodermal* parameters, no multivariate effect of introduction format was shown (Wilks’ Lambda = 0.99, *F*(2, 123) = 0.53, *p* = 0.588, ${\eta}_{partial}^2$= 0.01).

### The influence of introduction format and camera focus on the psychophysiological response during the video-vignette consultation

Multivariate *cardiovascular* results showed a medium effect of introduction format (Wilks’ Lambda = 0.95, *F*(3, 147) = 2.65, *p* = 0.051, ${\eta}_{partial}^2$= 0.05) when comparing cardiovascular activity during the consultation part of the vignette with baseline levels: the audiovisual introduction (format A) elicited a stronger cardiovascular response in analogue patients than the written introduction (format B). Follow up univariate results combined with graphical inspection (Fig. 3b) revealed that the audiovisual introduction caused a stronger systolic blood pressure response (*p* = 0.015, ${\eta}_{partial}^2$= 0.04) and diastolic blood pressure response (*p* = 0.015, ${\eta}_{partial}^2$= 0.04) compared to the written introduction. No effect on heart rate was shown (*p* = 0.314, ${\eta}_{partial}^2$< 0.01). No multivariate effects of camera focus (*p* = 0.187) nor an interaction effect between introduction format and camera focus (*p* = 0.180) were found on the *cardiovascular* response as evoked by the video-vignette consultation.

**Fig. 4:**
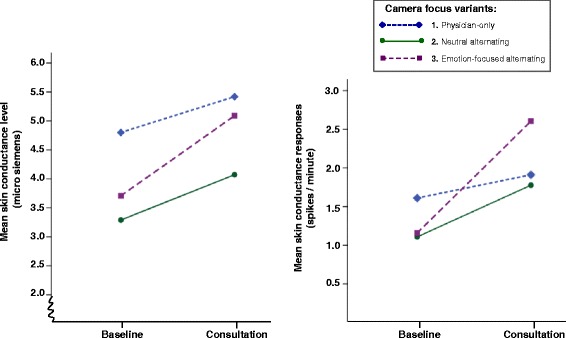
Impact of camera focus on the increase in analogue patients’ electrodermal activity from baseline to the video-vignette consultation. *Notes.* The graph on the left shows analogue patients’ mean skin conductance level and the graph on the right mean skin conductance responses, at baseline and during the video-vignette consultation, stratified by camera focus variants. Analogue patients who watched the vignette with emotion-focused alternating camera focus (variant 3) showed a larger increase in electrodermal activity compared to those who watched one of the other two variants (SCL: *p* = 0.005; SCRs: *p* = 0.001)

A multivariate medium effect of camera focus was found when comparing analogue patients’ *electrodermal* activity levels during the consultation part of the vignette to their baseline activity levels (Wilks’ Lambda = 0.88, *F*(4, 240) = 4.08, *p* = 0.003, ${\eta}_{partial}^2$= 0.06). More specifically, graphical inspection (see Fig. 4) revealed that analogue patients who watched the vignette with emotion-focused alternating camera focus (variant 3) showed a larger increase in electrodermal activity compared to those who watched one of the other two variants, which was confirmed in the follow up univariate analyses (SCL: *p* = 0.005, ${\eta}_{partial}^2$= 0.08; SCRs: *p* = 0.001, ${\eta}_{partial}^2$= 0.11). No multivariate effect of introduction format (*p* = 0.142) nor an interaction effect between introduction format and camera focus (*p* = 0.260) were found on the *electrodermal* response as evoked by the video-vignette consultation.

### The influence of introduction format and camera focus on perceived realism

Additionally, the influence of introduction format and camera focus on perceived realism of the video vignette was explored. Analogue patients scored on average 5 or more (possible range 1–7) on all three perceived realism scales (Table 3). A medium multivariate effect was shown for camera focus (Wilks’ Lambda = 0.90, *F*(6, 344) = 3.15, *p* = 0.005, ${\eta}_{partial}^2$= 0.05). More specifically, univariate results showed an effect on perceived realism of the video patient only (*p* = 0.003, ${\eta}_{partial}^2$= 0.07); no effects on perceived realism of the video vignette or the physician were found (*p*-values > 0.725). Post-hoc tests revealed that analogue patients in the video-vignette conditions with the emotion-focused alternating camera focus (variant 3), perceived the video patient’s appearance and behavior as more realistic than those who watched the physician-only (*p* = 0.003) or the neutral alternating (*p* = 0.003) camera focus variants. No main effect of introduction format (*p* = 0.592) nor an interaction effect between introduction format and camera focus (*p* = 0.437) were found.

**Table 3:** Perceived realism of the video vignette, physician and patient: means and standard deviations stratified by introduction format (A or B) and camera focus (1, 2 or 3)

			Perceived realism of the		
Introduction format	Camera focus		vignette	physician	patient
		N	M (SD)	M (SD)	M (SD)
A. Audiovisual	1. Physician-only	30	5.52 (1.10)	5.63 (1.18)	4.98 (1.26)
	2. Neutral alternating	31	5.51 (1.18)	5.65 (1.05)	4.82 (1.38)
	3. Emotion-focused alternating	31	5.53 (0.96)	5.40 (1.17)	5.35 (1.42)
	Total	92	5.52 (1.07)	5.56 (1.13)	5.05 (1.36)
B. Written	1. Physician-only	29	5.64 (1.03)	5.47 (1.38)	4.45 (1.37)
	2. Neutral alternating	30	5.34 (1.31)	5.43 (1.26)	4.58 (1.50)
	3. Emotion-focused alternating	29	5.51 (0.85)	5.40 (1.17)	5.59 (1.25)
	Total	88	5.50 (1.08)	5.43 (1.26)	4.87 (1.46)
Total	1. Physician-only	59	5.58 (1.06)	5.55 (1.28)	**4.72** (1.33)
	2. Neutral alternating	61	5.43 (1.24)	5.54 (1.16)	**4.70** (1.43)
	3. Emotion-focused alternating	60	5.52 (0.90)	5.40 (1.16)	**5.47** (1.33)
	Total	180	5.51 (1.07)	5.50 (1.19)	4.96 (1.41)

*Notes*. Possible range in values is 1–7; higher values indicate higher levels of perceived realism. The **bold** values indicate a significant effect of camera focus: analogue patients who watched the video vignettes with the emotion-focused alternating camera focus (variant 3), perceived the video patient’s appearance and behavior as more realistic than those who watched the physician-only (*p* = 0.003) or the neutral alternating (*p* = 0.003) variants

## Discussion

Video-vignette studies are imperative for the systematic investigation of physician-patient communication. Results of this experimental study indicate that methodological choices with regard to video-vignette development can influence the ecological validity of video vignettes. First, how the video vignettes are introduced mattered: analogue patients showed stronger *emotional* engagement in response to an audiovisual introduction, showing the patient in the hospital’s waiting area while the story was introduced by a voice-over, as compared to a written introduction displayed on a computer screen. In particular, the audiovisual introduction caused a stronger cardiovascular response in analogue patients while viewing the video-vignette introduction. This effect was sustained during the subsequent watching of the medical consultation. Second, alternating camera focus between the physician and the patient, specifically depicting the patient’s emotions, caused the strongest *emotional* engagement in analogue patients, as reflected by their electrodermal response to the vignette. Alternating camera focus between the physician and the patient, depicting the patient at relatively neutral moments in the consultation, did not enhance engagement compared to showing only the physician, indicating that the effect on emotional engagement was indeed a result of showing the patient’s emotional facial expressions. Third, no effects were found on analogue patients’ self-reported engagement, with only one exception; an interaction effect of introduction format and camera focus was found on analogue patients’ self-reported *emotional* engagement. Focusing only on the physician during the consultation resulted in lower levels of emotional engagement when combined with the audiovisual introduction, as compared to the written introduction. This may indicate that analogue patients’ emotional engagement is disrupted by not showing the video patient during the video-vignette consultation if the video patient has been audio-visually introduced first. Fourth, exploratory analyses showed that the perceived realism of the video patient was influenced by the variation in camera focus. Findings suggest that analogue patients need to see, and not only hear, the patient during the video-vignette consultation, in particular at emotional moments, to perceive the patient and his/her behavior as realistic. Summarizing, our findings imply that an audiovisual video-vignette introduction combined with a camera focus on patient’s emotional expressions during the video-vignette consultation results in the highest levels of *emotional* engagement in analogue patients. The magnitude of effects found on analogue patients’ emotional engagement, in particular on the psychophysiological measures (which were medium effects), were comparable to the effects of analogue patients’ age [12], and physician’s trust-conveying [22] or affective [23] communication, as shown in previous video-vignette studies. This underscores the significance of current findings. This evidence may inform methodological decisions during the development of video vignettes, thereby enhancing the ecological validity of future video-vignettes studies.

The current findings correspond with previous research showing an increase in electrodermal activity and blood pressure in response to video clips in which the characters express emotions, such as sadness and fearfulness [24]. This increase in physiological activity is most often interpreted as emotional arousal, but other psychological processes, such as attention can also lead to changes in physiological activity [11, 14]. To establish whether these effects signify emotional arousal or attention, results for self-reported engagement can be examined. However, in this study no direct effects of introduction format and camera focus were found on self-reported engagement. Nevertheless, the interaction effect on self-reported emotional, and not attentional, engagement implies that the increase in physiological activity may be best interpreted as reflecting analogue patients’ emotional engagement. Moreover, these results indicate that neither analogue patients’ identification with the video patient, nor their attention to, and immersion in the video vignette, are influenced by introduction format or camera focus. Other methodological considerations, such as video duration, age of analogue patients [12] or the casting of actors, might be more relevant to these dimensions of engagement, and should be investigated in future research.

Patient emotions are an inherent part of most medical consultations and therefore inducing emotional engagement, including emotions, in analogue patients is desirable. Based on the current findings, we would thus advise to enhance analogue patients’ emotional engagement by introducing the video vignette audio-visually and by alternating camera focus, depicting the video patient’s emotions. Nevertheless, this rationale requires some qualification. First, inducing particularly strong emotions may be less desirable for research in which emotions might distract analogue patients from the task at hand, e.g., the evaluation of the physicians’ information provision [2]. The current results indicate that limiting emotional engagement is feasible if necessary for specific study purposes, without heavily impacting other dimensions of engagement. Second, analogue patients may be steered or biased towards experiencing particular emotions as a consequence of observing the emotional response of the video patient. Focusing on the video patient’s emotional expressions might therefore not *always* be the best option. Researchers who do not want to show the patient at all during the video-vignette consultation should consider using a written introduction instead of an audiovisual introduction. Alternating camera focus between physician and patient showing the patient at relatively neutral moments during the medical interaction might be a suitable alternative as well.

A strength of the current study is its combined use of a multidimensional self-reported measure of engagement and physiological measures indicating emotional engagement. Previous research using video vignettes has shown limited overlap between these measures [12], which underscores their unique value. The current results suggest that the psychophysiological measures may have been more sensitive to the effects of variations in the methodological aspects under investigation. Furthermore, the use of psychophysiological measures enabled disentangling the effects of the methodological variations in specific phases of the video vignette. A limitation of these psychophysiological measures is their lack of specificity in meaning. The effects on electrodermal and cardiovascular measures in this study indicate increased involvement of the sympathetic, and/or the withdrawal of the parasympathetic branches of the autonomic nervous system [11]. As emotional and attentional processes activate the same bodily systems, this may signify either emotion or attention or both [8]. At present, psychophysiological measures are most often used to indicate emotional arousal [25], without differentiating between emotions. However, in a review by Kreibig, it is argued that the emotional arousal response is more differentiated, with specific patterns of peripheral physiological activation in response to different emotions [14]. The differential effects found in this study, i.e., introduction format affecting cardiovascular but not electrodermal activity, and camera focus influencing electrodermal but not cardiovascular activity, may therefore indicate that introduction format and camera focus may affect different emotions. The current state of evidence is however not sufficient to further speculate on this matter and future research into this area is thus warranted. Another limitation regards our sample of analogue patients. Although our student sample enabled the recruitment of a large number of analogue patients and restricted the (possible) confounding influence of variables such as experience with disease or medical consultations, this sample strategy limits the generalizability of our results. For example, older individuals’ engagement is on average stronger [12], and therefore a more heterogeneously aged sample might enhance variability in engagement levels and make it more likely to find effects. Furthermore, this study compared the effects of two introduction formats and three camera focus variants, because these variations were often used in video-vignette studies [2] and/or were expected to affect engagement differently. However, other variations in introduction and camera focus are possible, e.g., a read out loud introduction by the researcher [26], and should be investigated in future research.

## Conclusions

In sum, researchers in medical communication should carefully consider how they introduce their video vignettes and which camera focus they choose, as these decisions may influence analogue patients’ engagement with the video vignette and perceived realism of the video patient. Using an audiovisual introduction and alternating camera shots focused on the video patient’s emotional expressions during the medical consultation maximizes emotional engagement, and could therefore increase the ecological validity of the video-vignettes design. This study thereby contributes to the evidence base for making methodological choices in the development of experimental, video-based research on medical communication. This will yield more robust findings on the impact of physician-patient communication on patient outcomes that can be employed to improve medical care.

## Abbreviations

DBP: Diastolic blood pressure
HR: Heart rate
SBP: Systolic blood pressure
SCL: Skin conductance level
SCRs: Skin conductance responses
